# A Prioritized Patient-Centered Research Agenda to Reduce Disparities in Telehealth Uptake: Results from a National Consensus Conference

**DOI:** 10.1089/tmr.2023.0051

**Published:** 2023-12-29

**Authors:** Kristin L. Rising, Mackenzie Kemp, Amy E. Leader, Anna Marie Chang, Andrew J. Monick, Amanda Guth, Tracy Esteves Camacho, Gregory Laynor, Brooke Worster

**Affiliations:** ^1^ Department of Emergency Medicine, Thomas Jefferson University, Philadelphia, Pennsylvania, USA.; ^2^ Jefferson Center for Connected Care, Thomas Jefferson University, Philadelphia, Pennsylvania, USA.; ^3^ Division of Population Science, Department of Medical Oncology, Thomas Jefferson University, Philadelphia, Pennsylvania, USA.; ^4^ Sidney Kimmel Medical College, Thomas Jefferson University, Philadelphia, Pennsylvania, USA.; ^5^ Health Sciences Library, NYU Grossman School of Medicine, New York, New York, USA.; ^6^ Department of Medical Oncology, Jefferson Health, Philadelphia, Pennsylvania, USA.

**Keywords:** telemedicine, health care disparities, health care quality, access and evaluation

## Abstract

**Introduction:**

We hosted a national consensus conference with a diverse group of stakeholders to develop a patient-centered research agenda focused on reducing disparities in telehealth use.

**Methods:**

Attendees were purposively invited to participate in a 2-day virtual conference. The group developed a prioritized research agenda focused on reducing disparities in telehealth uptake, with discussion informed by findings from a scoping review. All work was conducted in partnership with a Steering Committee of national experts in telehealth and patient-centered care (*n* = 5) and a community-based Telehealth Advisory Board with experience with telehealth use and barriers (*n* = 8).

**Results:**

Sixty individuals participated in the conference and discussion resulted in a final list of 20 questions. Fifty-two attendees voted on the final prioritization of these questions. Results were aggregated for all voters (*n* = 52) and patient-only voters (*n* = 8). The top question identified by both groups focused on patient and family perspectives on important barriers to telehealth use. The entire group voting identified telehealth's impact on patient outcomes as the next most important questions, while the patient-only group identified trust-related considerations and cultural factors impacting telehealth use as next priorities.

**Conclusions:**

This project involved extensive patient and stakeholder engagement. While voting varied between patients only and the entire group of conference attendees, top identified priorities included patient and family perspectives on important barriers to telehealth, trust and cultural barriers and facilitators to telehealth, and assessment of telehealth's impact on patient outcomes. This research agenda can inform design of future research focused on addressing disparities in telehealth use.

## Introduction

The COVID-19 pandemic exacerbated health and social inequities. Patients were more likely to avoid seeking necessary health care, and providers were forced to triage treatment by urgency due to resource constraints.^1^ Telehealth use surged during the pandemic to facilitate patient access to needed health care while minimizing risk of COVID-19 exposure.^2,3^ Telehealth connects individuals to their health care providers when in-person care is not available, necessary, or possible.^4^ Modes of telehealth may include talking to a provider over the phone or video chat, sending and receiving messages using a secure portal, or using a remote monitoring device to share health data with a provider. Services are typically categorized as synchronous (allowing for direct, real-time engagement between patient and provider) or asynchronous (nondirect communication, like storing and forwarding information).

While this growth in telehealth use facilitated access to needed care for many patients, it also threatened to worsen an already significant disparity in outcomes for vulnerable populations. Factors that impact patient access to and use of telehealth are complex. The American Telemedicine Association describes 10 related components or elements that are needed to eliminate health disparities using telehealth, ranging from structural anti-racism to connectivity.^5^

Digital readiness is a term that encompasses the digital skills, trust, and ability to use tools needed to carry out online tasks.^6^ Approximately 29% of Americans have low levels of digital readiness, which transcends lack of access to digital devices; for example, lack of readiness may be driven by low trust in digital health platforms or lack of skills to navigate digital health platforms.^7^ Digital readiness barriers can lead to disparities in engagement with telehealth-based clinical care.^8^ Left unaddressed, disparities in telehealth use and uptake will exacerbate gaps in access to evidence-based tools and resources and lead to increased health disparities.^5^

To that end, our team undertook a two-step process to develop a patient-centered research agenda to reduce disparities in telehealth uptake. This process was conducted in close partnership with a Philadelphia-based patient advisory board and a national Steering Committee comprised of experts in patient communication, patient and family centered care, telehealth, and health care disparities. In the first step, we performed a scoping review to understand the current state of literature regarding both barriers and disparities in telehealth uptake. In the second step, we held a national consensus conference in which we convened a broad range of stakeholders to review the results of the scoping review and to use findings to develop a prioritized research agenda. In the following article, we report on these activities, including a list of the final prioritized research agenda.

## Methods

Conference planning was initiated with the establishment of two stakeholder advisory groups, one national and one local. The national Steering Committee was convened in August 2021 to support both the scoping review and consensus conference. Committee members had expertise in patient-centered care, patient and family advocacy, health care equity and disparities, and telehealth practice, policy, and research. Five members participated in meetings over a 17-month period to inform the development of scoping review research questions and data extraction tools and to support the planning, implementation, and reporting of the consensus conference. As this was not human subjects research, IRB approval was not required.

In addition, patients and community members in the Philadelphia region were invited in October 2021 to join a Telehealth Advisory Board (TAB) to inform patient-centered consensus conference planning and implementation. Members were primarily recruited through local community organizations and existing patient advisory councils, with the goal of convening a diverse set of stakeholders who represent various communities impacted by telehealth disparities in the greater Philadelphia region. Eight members participated in monthly meetings to inform conference invitations, agenda, materials, and patient engagement, and all TAB members were invited to the consensus conference. Two TAB members cochaired the group and were primarily responsible for meeting facilitation.

A major first step of conference planning included a scoping review to answer the question: What barriers and disparities in telehealth uptake and use have been documented in the literature? The primary purpose of the scoping review was to inform conference discussion and consensus building. The review was undertaken in accordance with the Joanna Briggs Institute (JBI) methodological guide for scoping reviews.^9^ The scoping review process was developed collaboratively between the project team, a medical librarian, and the steering committee. For the purpose of the review, the team used the following operational definitions:

- Telehealth: Bidirectional communication between a patient and a provider. Includes both synchronous (communication occurring between provider and patient at the same time, such as a video visit) and asynchronous (communication occurring at different times, such as text messages or e-mails).
- Barrier: A factor (experiences or perceptions) that negatively impacts patients' telehealth use.
- Disparity: A difference in telehealth use between two groups that is found to be statistically significant (*p* < 0.05).

While the scoping review included literature published globally between 2011 and 2021, data presented at the consensus conference narrowed in on sources from the United States published between 2017 and 2021 to help focus discussions. The scoping review protocol was submitted with Open Science Framework registries (https://osf.io/df6aw/). Comprehensive scoping review results will be published in a forthcoming manuscript.

The national consensus conference was held from September 28–29, 2022, via Zoom. The purpose was to develop a patient-centered prioritized research agenda focused on reducing disparities in telehealth uptake and use. The consensus conference convened a broad group of stakeholders (*n* = 60) from across the country with experience and expertise in telehealth infrastructure, policy, and patient care. Attendees were selected for their experience and engagement with telehealth. Both the TAB and steering committee provided input on conference invitations. In alignment with recommendations from the JBI Scoping Review Methodology Group, attendees also represented knowledge users relevant for the interpretation of scoping review results.^10^ A full list of organizations represented by attendees can be found in Table 1.

**Table 1.** Conference Attendees

Conference attendee organization/affiliation
• AHIMA Foundation (*n* = 1)
• AIRnyc (*n* = 1)
• Association of American Medical Colleges (*n* = 1)
• ATW Health Solutions (*n* = 1)
• California Health Care Foundation (*n* = 1)
• Cambridge Health Alliance (*n* = 1)
• Center for Care Innovations (*n* = 1)
• Children's Hospital Colorado (*n* = 1)
• Children's Hospital New Orleans (*n* = 1)
• Children's National Hospital (*n* = 1)
• ChristianaCare (*n* = 1)
• Cityblock Health (*n* = 1)
• CityLife Health (*n* = 1)
• Community Care Cooperative (C3; *n* = 1)
• CSL Behring (*n* = 1)
• Dalhousie University, Canada (*n* = 1)
• Massachusetts General Hospital (*n* = 1)
• Duquesne University Center for Healthcare Ethics (*n* = 1)
• Global Patient and Family Advisory Board (*n* = 1)
• Great Plains Telehealth Resource and Assistance Center (*n* = 1)
• George Washington University Medical Faculty Associates (*n* = 1)
• Hassanah Consulting (*n* = 1)
• Health Partners Plans (*n* = 1)
• HonorHealth (*n* = 1)
• Institute for Patient- and Family-Centered Care (*n* = 1)
• Jefferson Health (*n* = 4)
• Kennedy Krieger Institute (*n* = 1)
• Laurel Health Advisors, LLC (*n* = 1)
• Massachusetts General Hospital (*n* = 2)
• Microsoft Corporation (*n* = 1)
• Mid-Atlantic Telehealth Resource Center/University of Virginia School of Medicine (*n* = 2)
• Mount Sinai Health System (*n* = 1)
• Mythical Beast Consulting (*n* = 1)
• National Association of Community Health Centers (*n* = 1)
• Nest Health (*n* = 1)
• New York University School of Medicine/NYU Langone Health System (*n* = 1)
• Philadelphia Department of Public Health (*n* = 2)
• Press Ganey Associates (*n* = 1)
• Primary Health Care Inc. (*n* = 1)
• RAND Corporation (*n* = 1)
• Southeast Asian Mutual Assistance Association Coalition (*n* = 1)
• Southwest Telehealth Resource Center (*n* = 1)
• Stanford University, Roots Community Health Center (*n* = 1)
• State Council for Persons with Disabilities (*n* = 1)
• Team Josiah 2K22 Foundation Inc. (*n* = 1)
• Telehealth Advisory Board Member (*n* = 2)
• UNC Health (*n* = 1)
• University of California San Francisco (*n* = 1)
• University of Kansas Medical Center (*n* = 1)
• University of Missouri, School of Medicine (*n* = 1)
• Uriel E. Owens Sickle Cell Disease Association of the Midwest (*n* = 1)
• West Health Institute (*n* = 1)

The conference began with an introduction by project lead Dr. Kristin Rising, in which an overview of the scoping review process and conference purpose were shared. Next, Dr. Amy Leader presented the scoping review findings relevant to “barriers.” Attendees were then divided across six breakout groups to discuss gaps in the research and draft potential research questions related to “barriers.” After small group discussion, attendees came back together into the full group and a member of each breakout group presented key discussion points and the list of generated questions to the full group.

During a break, the project team compiled draft research questions across all breakout groups and entered them into Qualtrics online survey platform.^11^ Each attendee then voted on the level of importance of the entire list of drafted questions using the online voting platform. The survey for each question read, “As an area of research, the following question is: 1) not important, 2) somewhat important, 3) neutral, 4) important, or 5) very important?” Attendees were also asked to self-identify as an academic, clinician, community representative, industry representative, or patient to allow for sorting of voting results by different groups. All 60 external attendees as well as the three project leads (K.L.R., A.E.L., B.W.) were invited to vote.

Upon completion of discussion and voting on “barriers,” Dr. Brooke Worster presented the scoping review findings and draft research questions regarding “disparities” to the entire conference group. The same processes for breakout groups and voting were then repeated for discussion related to disparities in telehealth uptake. At the end of the 1st day, there were 58 newly drafted and voted on research questions, 35 of which came from the “barriers” discussion and 23 from the “disparities” discussion. A total of 56 attendees submitted a voting survey on day 1, including the three project leads. A full list of questions, with the original language maintained, can be found in Table 2.

**Table 2.** Attendee Generated Draft Research Questions

Barrier-related questions
1. What is the patient perspective of important barriers to telehealth?
2. Does mitigating the disparities in TH use increase health outcomes? Which outcomes?
3. What interventions are most effective at reducing each of the identified barriers?
4. What infrastructure and support (i.e., the processes) are needed for patients and providers to successfully complete TH visits? How does this vary by populations? What processes and support is needed for follow-up and continuing care?
5. What is an effective way to facilitate access to and use of telehealth?
6. Does addressing specific barriers to telehealth use actually result in increased telehealth use and improvement in other patient-important outcomes?
7. What is the best approach to increase patient trust in telehealth?
8. What are the factors at the organizational level that serve as barriers to telehealth?
9. Does addressing any of the individual barriers actually increase access to high quality coordinated care for disadvantaged populations?
10. What cultural factors (and trust-related factors) serve as barriers to telehealth?
11. How can health systems and community organizations partner to address barriers?
12. How do we educate providers at all levels to use telehealth?
13. How can health systems and community organizations partner to address barriers?
14. Can community health workers/peers effectively address barriers to telehealth?
15. Are interventions effective across various populations?
16. Which telehealth interventions are the most sustainable?
17. What are the necessary and sufficient conditions to enable access to telehealth?
18. Which interventions give patients skills that are transferrable/generalizable across a range of settings?
19. How can multisector partnerships be built and most effectively collaborate to support telehealth use?
20. What is an efficient way to screen across a population to assess the most common or pressing barriers to telehealth?
21. Who is most effective in teaching how to build trust and communicate effectively in telehealth? Are patients and family representatives helpful with this?
22. Does using any of the identified facilitators actually increase access to telehealth?
23. When is TH appropriate/not appropriate?
24. How has telehealth been beneficial to patients seeking mental health care? In what outcomes?
25. How do we educate providers at all levels to use TH with patients? Where in educational curricula is TH taught?
26. Is the quality of engagement and communication equally as good on telehealth as it is in person?
27. Who is best equipped to implement interventions for these barriers?
28. What other ways is telehealth beneficial at a population level?
29. Which interventions are easiest to implement?
30. Is there a cost advantage to TH, in terms of costs and staffing?
31. Is it possible to develop a digital literacy standard for telehealth usability?
32. What success stories do we have that shows that TH is making a difference?
33. Where in the educational curricula should TH be taught?
34. Does TH increase or decrease emergency department visits?
35. Is a particular intervention focused on using a particular platform generalizable across platforms/settings?
**Disparity-related questions**
1. What outcomes for measuring telehealth success are most important to patients?
2. How can we leverage telehealth to reach those without access and promote future engagement in hybrid care?
3. What are the appropriate metrics for assessing success in telehealth interventions, other than use/nonuse?
4. What accommodations do people with various disabilities need to engage in telehealth?
5. What are the preferences of the patient, and how are these preferences being used/considered by providers?
6. What provider-level (e.g., language concordance) and practice-level (e.g., digital navigator presence) factors can reduce disparities in TH use?
7. To what extent do documented disparities represent actual disparities unique to telehealth vs. actual more pervasive disparities in health care and technology?
8. What commonly held assumptions about barriers and disparities are continuing to contribute to patient disparities in telehealth use?
9. What are the drivers of disparities in specific populations (payers, equipment, SES of patients)?
10. Do disparities in access by type of TH (e.g., synchronous vs. asynchronous, audio vs. video) lead to differences in outcomes?
11. Are there certain telehealth models (e.g., hosted in clinic, at home, at community site) that can reduce disparities in use?
12. How can we redefine costs to better assess longitudinal return on investment of telehealth?
13. How can we teach an unbiased approach to delivery of telehealth in medical education (both didactic and in clinical training)?
14. How do we differentiate between telehealth modalities and the decision of when to use one versus another?
15. How can we systematically assess and address the unique experiences of individuals instead of relying on common group categories?
16. What system-level choices about telehealth products may contribute to patient TH use?
17. Do patients feel a greater sense of agency or confidence with use of telehealth?
18. Where do patient-preferred and health system-preferred outcomes overlap and how do we reconcile for a given study if they don't overlap? (e.g., is readmission rate a good outcome since it has major health system value, but might devalue patient preference for earlier discharge)
19. Are there differences in access to TH in the various types of TH?
20. How do we support patient access to telehealth without them feeling pushed into telehealth?
21. Who has the most agency/ability to facilitate access to telehealth?
22. At what level of society is the responsibility or ability to address various disparities?
23. What barriers have been created (by patient and provider alike) leading us to believe that certain medical specialties are better primed to offer telehealth?

SES = socioeconomic status; TH = telehealth.

In preparation for day 2, the research team compiled voting results across all 58 questions to identify the top-rated questions for discussion. Questions were sorted by mean score and the top 20 questions from the full group voting results as well as patient-only results were extracted. Any questions that were included in either (1) the top 10 rating from either group or (2) the top 20 rating in both groups were kept in the list for discussion on day 2. This resulted in 17 questions for group discussion in day 2 (Table 3).

**Table 3.** Top-Rated Research Questions

Barriers
1. What is the patient perspective of important barriers to telehealth?
2. What cultural and trust-related factors serve as barriers to telehealth?
3. What organization-level factors serve as barriers to telehealth?
Trust
4. What is the best approach to increase patient trust in telehealth?
5. Who is most effective in teaching how to build trust and communicate effectively in telehealth? Are patients and family representatives helpful with this?
6. Can community health workers/peers effectively address barriers to telehealth?
Outcomes
7. Does mitigating disparities in telehealth use improve health outcomes? Which outcomes?
8. Does addressing specific barriers to telehealth use actually result in increased telehealth use and improvement in other patient-important outcomes?
9. Does addressing any of the individual barriers increase access to high-quality coordinated care for disadvantaged populations?
10. How has telehealth been beneficial to patients seeking mental health care? In what outcomes?
Interventions/infrastructure
11. What interventions are most effective at reducing each of the identified barriers?
12. What is an effective way to facilitate access to and use of telehealth?
13. What infrastructure and support (i.e., the processes) are needed for patients and providers to successfully complete TH visits? How does this vary by populations?
14. Which interventions give patients skills that are transferrable/generalizable across a range of settings?
15. How do we educate providers at all levels to use telehealth?
16. How can health systems and community organizations partner to address barriers?
17. How can multisector partnerships be built and most effectively collaborate to support telehealth use?

Discussion on the 2nd day started with a review of the top 17 questions identified based on day 1 voting. Upon presentation of the questions, conference attendees identified challenges in comparing the individual questions due to variable wording and level of focus across the questions. The group collectively decided that it would be more informative to identify priority research themes that represented the questions, and then develop a final set of focused questions later. The remainder of the discussion on day 2 was spent identifying 15 research themes that were organized into 4 categories, with some overlap in content: barriers, trust, outcomes, and interventions/infrastructure (Table 4).

**Table 4.** Priority Research Themes and Categories

Category	Themes
Barriers	• Patient-important barriers and facilitators to telehealth • Cultural barriers and facilitators to telehealth • Trust-related barriers and facilitators to telehealth • Linguistic challenges including interpersonal technological • Technology barriers for disabled individuals • Screening approaches to facilitate individualized care
Trust	• Patient trust/mistrust in telehealth • Trusted messengers and support people
Outcomes	• Impact of interventions to facilitate telehealth on patient outcomes and value of care • Patient-important outcomes related to telehealth (includes quality, meeting needs of the family)
Interventions/infrastructure	• Infrastructure and support needed for patients and providers • Addressing structural and systemic inequities • Patient-centered provider and team education on use of telehealth • Effective interventions to facilitate access to telehealth • Multisector partnerships to support telehealth equity

After conclusion of the conference, the research team developed a set of research questions to represent the key categories and associated research themes identified during the conference. These questions were iteratively refined by the TAB and the Steering Committee members to ensure that the content adequately reflected the conference findings, and that the question wording was clear and balanced across questions. A final list of 20 questions was sent out to all attendees electronically for a final round of voting, which focused on attendees developing a rank order of questions from most to least important. Voting results were discussed with both the TAB and the Steering Committee, with a focus on exploring potential causes or meaning of the most significant voting discrepancies.

## Results

### Scoping review

The database searches yielded 11,156 citations after deduplication. Title and abstract screening resulted in the exclusion of 9,913 citations that did not meet identified inclusion criteria. A total of 1,243 full-text citations were assessed for eligibility. Upon full-text review, an additional 623 citations were excluded for various reasons. There were 618 references ultimately included for data extraction.

Sixty-four percent (*n* = 394) of all of the sources were based in the United States, while the remaining studies took place in numerous other countries. A total of 369 sources (60%) were published between 2020 and 2021, representing a significant surge in telehealth-related publications during the years of COVID-19. A majority of sources looked at synchronous modalities of telehealth (69%, *n* = 430) and collected data from the perspective of patients and their families (85%, *n* = 526). More studies utilized quantitative methods (57%, *n* = 354) than qualitative methods (28%, *n* = 171) or mixed methods (15%, *n* = 91). A total of 428 sources to 395 sources (64%) included data on barriers to telehealth uptake and use and 293 sources (47%) pertained to telehealth disparities.

### Conference attendees

During conference registration, attendees were asked to indicate which professional group they most identified with. Sixteen individuals identified as clinicians (27%), 12 as nonclinician academics (20%), 8 as patients (13%), 8 as industry representatives (13%), 7 as community organization representatives (12%), and 9 as other (15%).

### Conference breakout group discussions

Across the six barrier-focused breakout rooms, groups commonly discussed the relative importance of access and outcomes. They worried that removing barriers might not, as assumed, lead to increased telehealth uptake. Many participants also noted that barriers were framed through patient differences (e.g., race, age) rather than the underlying structural causes. They thought about whether barriers transcended individual factors and might better be considered at the system level. All groups were in favor of using digital navigators to reduce barriers. Several groups considered the implicit assumption that video telehealth is preferred to audio-only as well as remarking upon provider education and training as impediments to patient uptake of telehealth.

Across the disparity-focused breakout groups, the underlying premise that disparities arose due to deeper societal flaws rather than demographic characteristics was ubiquitous. A common thread between groups was how telehealth must be tailored to address the needs of different populations—both to drive access and to deliver care equitably. Most groups identified that measures of success in reducing disparities should be patient-centered and patient-identified, rather than assumed by researchers.

### Voting results

A total of 52 conference attendees voted on the final list of research questions (86.7% response rate). As the focus of this conference was to establish a patient-centered prioritized research agenda, voting results were assessed for the full group of attendees as well as for the patient-only group. While the top question was the same across both groups, differences arose among lower-priority items. Table 5 shows the 20 questions and voting results.

**Table 5.** Final List of Research Questions as Prioritized By All Voters (*n* = 52) and Patient-Only Voters (*n* = 8)

Question	Full group rank (***n*** = 52)	Patient-only rank (***n*** = 8)
1. What facilitators and barriers to telehealth use are most important to address from the patient and family perspective?	1	1
2. Does facilitating telehealth use improve health outcomes among marginalized populations?	2	12
3. What are mechanisms by which telehealth improves patient outcomes?	3	4
4. What are effective approaches to assessing the most common or pressing barriers to telehealth uptake among different populations?	4	7
5. What are the patient and family trust-related facilitators and barriers to telehealth use?	5	2
6. What are the necessary and sufficient conditions at each level (system, provider, patient, family) to facilitate access to telehealth?	6	16
7. What are the outcomes most important to patients and families for assessing the impact of interventions designed to increase telehealth use?	7	15
8. What are cultural facilitators and barriers to telehealth use?	8	3
9. What systemic/infrastructure changes have been implemented to enhance telehealth use, and what have we learned from them?	9	11
10. What linguistic interpersonal and technological challenges exist with telehealth use?	10	10
11. What are the outcomes most important to patients and families for assessing the impact of telehealth use?	11	5
12. What facilitators and barriers are most important to address in support of use of telehealth among individuals with disabilities and/or neurodiversity?	12	14
13. What approaches are most effective at improving patient and family trust in telehealth?	13	8
14. What interventions designed to address barriers in telehealth uptake are most effective at improving outcomes most important to patients and families?	14	19
15. What interventions are most effective at facilitating access to and use of telehealth?	15	20
16. How can multisector (health systems, community organizations, others) partnerships be built and most effectively collaborate to support telehealth use among disadvantaged populations?	16	9
17. How can interventions be efficiently and effectively deployed to provide personalized support to patients and families for addressing their unique combination of barriers to telehealth use?	17	18
18. What are best practices for providing patient-centered education to providers and team members of all departments in use of telehealth?	18	13
19. What success stories do we have that show telehealth is making a difference?	19	6
20. Who is most effective in providing assistance in telehealth use and building trust among patients, families and communities?	20	17

Questions with a difference >5 in rating between the two groups are shaded in grey.

Both TAB and Steering Committee members identified the fact that the questions ranked most highly by the full group were more focused on assessing outcomes related to telehealth use, while the questions ranked more highly by the patient-only group were more focused on facilitating telehealth use. One patient in the TAB identified the full group votes as “putting the cart before the horse,” suggesting that more attention was needed to understanding and addressing barriers to uptake before looking at outcomes. There was also discussion about the wording used to describe various populations in need, with members of the TAB identifying “marginalized” (as used in question 2) as a word that may be poorly understood or not relatable by patients. The TAB suggested that “disadvantaged” (as used in question 16) is a term that may resonate better with patients. In addition, rewording was suggested to improve question 19, as follows: “How do we use stories to best support telehealth and its impact?”

## Discussion

In this work, we conducted a scoping review to identify barriers to and disparities in telehealth uptake among various populations. We then convened a broad range of patients and stakeholders to develop a list of patient-centered research questions focused on understanding and reducing disparities in telehealth use. The selected questions were prioritized by all conference attendees, with voting results presented both for all attendees and exclusively patient attendees. All this work was conducted in close partnership with patient and community advisors to ensure patient centeredness of study conduct and findings.

The primary question as identified by all attendees and the patient attendee subgroup was “What facilitators and barriers to telehealth use are most important to address from the patient and family perspective?” Consideration of this question is vital to inform allocation of funding to support digital health equity. For instance, considerable funding has been allocated over the past few years to address limited technology and internet access, such as the COVID-19 Telehealth Program administered by the Federal Communications Commission. While these programs are intended to support the provision of devices to patients to increase engagement in telehealth, we lack data to confirm that increasing access to devices is in fact a primary priority of patients and families. Though all individuals may not have access to a device, this lack of access may not be seen as a primary issue to telehealth use until higher-order barriers, such as trust in use of telehealth or having privacy to engage in telehealth visits, are addressed. The voting results from this conference suggest the importance of engaging directly with patients and families in various communities to determine most effective allocation of future funding.

While there was agreement on the top priority question among all attendees and the patient subgroup, there were also differences in prioritization. Patients placed more emphasis on trust and cultural factors that affect telehealth uptake and use, whereas professionals prioritized health system and insurance operational barriers. Patients' priority on trust emphasized the importance of expanding conversations regarding telehealth disparities to consider barriers beyond solely “digital literacy.” When considering telehealth-specific uptake, our team has adopted the term “digital health readiness” as its overarching concept, which includes skills, trust, and use of digital tools.^6^ This dichotomy between patient and professional priorities is not only interesting but also vital to focus future research funding. Balancing both the systemic function and the human factors promoting and preventing telehealth utilization are the critical “next steps” in telehealth research.

This work provides valuable findings, yet it does have limitations. Regarding the scoping review, we purposefully kept the review very broad to ensure that all relevant texts were included. This meant, however, that the depth of findings presented at the conference was still very high level, as there was too much content to summarize in depth considering the bandwidth of the team and the time allotted to this task. Further work is needed to explore subtopics in greater depth within the scoping review articles. For the conference, while we strove to engage a diverse group, it was not possible to identify and include all potentially relevant populations among our conference attendees, and thus some voices were undoubtedly missed. In addition, while we had the opportunity to explore differences in voting outcomes between the patients and entire group of attendees with both our Steering Committee and TAB members, we lacked opportunity to elicit perspective on these outcomes from many of the conference attendees.

## Conclusion

The results from this work provide an important framework for use by both researchers and policymakers. The priorities we have established should help determine what next projects are most likely to advance our ability to understand and address disparities in telehealth uptake. Continued partnership with patients and community stakeholders is essential to ensure that the patient perspective continues to be incorporated into the design, implementation, and interpretation of future work, with the goal of building a more patient-centered care delivery system in which telehealth is a core component that all are able to access.

## Abbreviations Used

JBI: Joanna Briggs Institute
TAB: Telehealth Advisory Board
